# Hyperspectral light sheet microscopy

**DOI:** 10.1038/ncomms8990

**Published:** 2015-09-02

**Authors:** Wiebke Jahr, Benjamin Schmid, Christopher Schmied, Florian O. Fahrbach, Jan Huisken

**Affiliations:** 1 Max Planck Institute of Molecular Cell Biology and Genetics, Pfotenhauer Strasse 108, 01307 Dresden, Germany; 2 DIGS-BB, Technische Universität Dresden, Pfotenhauer Str. 108, 01307 Dresden, Germany

## Abstract

To study the development and interactions of cells and tissues, multiple fluorescent markers need to be imaged efficiently in a single living organism. Instead of acquiring individual colours sequentially with filters, we created a platform based on line-scanning light sheet microscopy to record the entire spectrum for each pixel in a three-dimensional volume. We evaluated data sets with varying spectral sampling and determined the optimal channel width to be around 5 nm. With the help of these data sets, we show that our setup outperforms filter-based approaches with regard to image quality and discrimination of fluorophores. By spectral unmixing we resolved overlapping fluorophores with up to nanometre resolution and removed autofluorescence in zebrafish and fruit fly embryos.

## Abstract (web summary)

Multicolour information is required to study the complex interplay of biological tissues. Here, Jahr *et al.* acquire spectral information at high resolution for each pixel in a hyperspectral light sheet microscope, while maintaining its perpendicular illumination and low phototoxicity.

## Graphical abstract

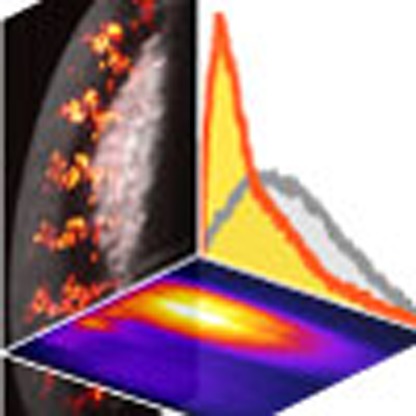

In fluorescence microscopy, differently coloured fluorophores are used to selectively label structures within tissues and to study tissue interactions. For *in vivo* microscopy, it is desirable to acquire many such colours efficiently during one recording within a single sample. Commonly, multiple colours are either imaged sequentially using different filter sets or split onto several cameras. Invariantly, both approaches rely on filters and are therefore ultimately limited by the spectral overlap of fluorescent markers, which makes it difficult in practice to distinguish more than three or four colours within the visible spectrum^1^. Information about the interaction of more components can only be obtained from consecutive experiments in different samples, which may exhibit strong variance. As an alternative, hyperspectral approaches have been developed in confocal microscopy, where a diffraction grating or prism is used to acquire the full spectrum at each point of the image^2^.

Due to its slow point scanning, confocal microscopy is not ideally suited to image large samples. Acquisition speeds can be improved by parallelizing the illumination scheme and simultaneously scanning several points along a line. Line-scanning confocal microscopy has been combined with a diffractive element and a two-dimensional sensor to acquire spectral information, but existing implementations compromise the amount of spatial information acquired by scanning only four points at a time^3^ or acquiring only 64 pixel × 32 pixel^4^.

To study biological processes like embryogenesis over extended periods of time, selective plane illumination microscopy (SPIM^5^) has become the technique of choice due to its low phototoxicity^6^,^7^,^8^. It is desirable to find a technique that provides spectral information for each pixel while maintaining the perpendicular illumination scheme of SPIM. In snapshot spectral imaging techniques, the spectral data cube is rearranged into multiple two-dimensional elements, which are then imaged simultaneously on a single detector^9^. Snapshot techniques could in principle be combined with SPIM's wide-field detection, but require detector space in order to accommodate spectral data, thereby sacrificing spatial information. To date, no technique is available to record spectrally resolved data sets while maintaining the spatial information and minimal light dose necessary to image large developing samples.

Here, we present a hyperspectral light sheet microscope that acquires spectral information at high resolution for each pixel, while still providing the perpendicular illumination scheme and the low phototoxicity of SPIM.

## Results

### Descanned detection

In light sheet microscopy, the sample is illuminated in a single plane either with a static light sheet generated by cylindrical optics^5^ or by rapidly scanning a beam across the plane to form a virtual light sheet^10^. We developed our hyperspectral setup on the basis of a beam-scanning SPIM. Usually, in such a configuration, the illumination beam is swept through the sample and the image is formed by integrating the signal as it moves across the camera chip (DSLM^10^). This approach, however, is very inefficient, as only one line of the camera chip is illuminated at a time while the remaining area of the detector is not used. Therefore, we developed a descanned detection: Instead of sweeping the signal across the detector, a descanning mirror in the detection path always projected the signal onto the same position on the detector. The data were then collected line-by-line and we reconstructed an image by using the position of the illuminated line in the sample, which is known from the calibration of the scan mirrors (Supplementary Fig. 1). Furthermore, this method may offer the additional benefit of contrast enhancement through confocal line detection^11^,^12^,^13^.

### Hyperspectral acquisition

**Figure 1:**
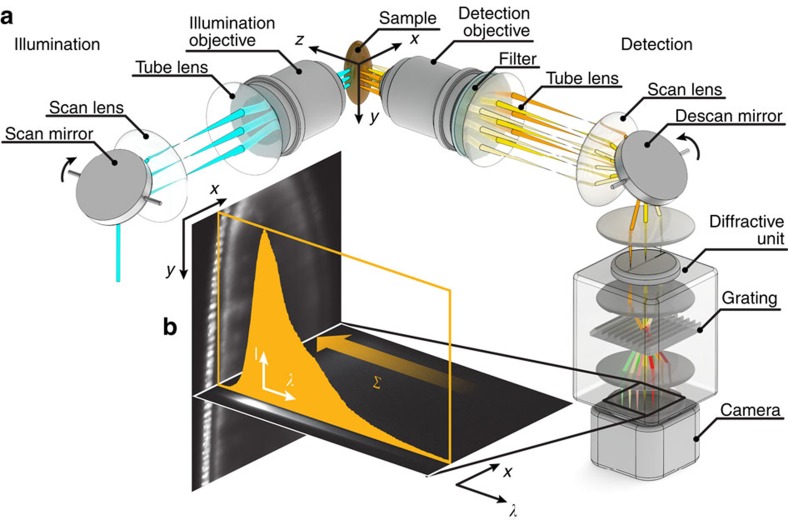
Hyperspectral SPIM setup and image formation. (**a**) Schematic of the setup. The light sheet is created by quickly scanning the illumination beam with a scan mirror. The sample is placed at the intersection of the focal planes of detection and illumination objective and illuminated with the scanned light sheet. The fluorescence signal is descanned onto a single line with a second scan mirror. A diffractive unit separates the spectral components of the incoming light spatially. (**b**) On the camera chip, a stack of *x*, *λ*-data sets was recorded, resulting in a three-dimensional data cube. Summing over all wavelengths (*Σ*) would yield the conventional, single-colour image. Here, a Drosophila embryo expressing histone-YFP in all cells is shown.

With our descanned detection, the image of the illuminated sample region no longer moved across the detector and remained in one position. Thus, only one detector dimension was needed for the spatial information, while the other was free to record spectral data. In order to separate the different wavelengths of the incoming multicolour signal spatially, we added a diffractive unit (Imspector V8E, Specim)^14^ to the detection path of our line-scanning SPIM (Fig. 1a and Supplementary Fig. 2). On the camera, *x*, *λ*-data sets were acquired for each *y* position in the sample (compare push-broom hyperspectral imaging^15^), resulting in a hyperspectral data cube (Fig. 1b). We altered the reconstruction algorithm accordingly: Instead of reconstructing a single-colour image, the recorded *y*-stack was now transformed into a *λ*-stack. The spectral response was calibrated by recording the signal of scattered laser light (Supplementary Fig. 3), delivering images with a spectral resolution of 0.5 nm per pixel.

As the fluorescence signal travelled through a number of optical elements in the beam path, each component altered the spectrum. To calculate the overall spectral sensitivity of our system and to correct the measured signal, we multiplied the quantum efficiency, transmission and reflection curves, respectively, for each optical element (Supplementary Fig. 2c,d). The corrected spectra for enhanced green fluorescent protein (EGFP) and mCherry were in good agreement with previous studies^16^ (Supplementary Fig. 2e). Our line-scanning SPIM with descanned detection and diffractive unit was able to record hyperspectral information with high spectral resolution and provided precise spectra across the entire visible range. Since the spectral sensitivity curve was fairly flat, no correction of the measured spectra was needed.

### Virtual filtering and linear unmixing of spectra

**Figure 2:**
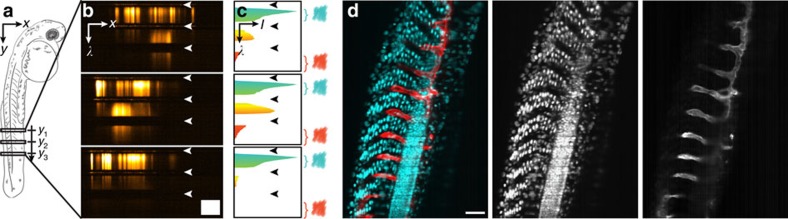
Image formation and virtual filtering. (**a**) Schematic drawing of a two days post fertilization zebrafish embryo imaged laterally. (**b**) Typical *x*, *λ*-data sets acquired along three different lines in a *Tg(h2afva:h2afva-GFP,kdrl:Hsa.HRAS-mCherry)* zebrafish embryo. Spatial information is displayed horizontally, spectral information vertically. Dark horizontal stripes (arrowheads) were caused by the QuadNotch filter blocking scattered illumination light. Scale bar, 100 nm (spectral) × 100 μm (spatial). (**c**) Spectra were extracted from the *x*, *λ*-data sets by integration along the *x* axis. From the spectra, regions that could be assigned unambiguously to a colour channel were chosen and integrated (‘virtual filtering'). (**d**) Reconstruction of a dual channel image with cyan (left): 475–555 nm, red (right): 601–718 nm. Scale bar, 100 μm.

To demonstrate our system's suitability for imaging living samples, we acquired and reconstructed *λ*-stacks of double fluorescently labelled zebrafish embryos, *Tg(h2afva:h2afva-GFP, kdrl:Hsa.HRAS-mCherry)* (Fig. 2). For fluorophores with little spectral overlap, such as EGFP and mCherry, filters are commonly used to distinguish between colours. We integrated the signal across each fluorophore band to extract dual-colour images from the *λ*-stack (Fig. 2b–d). We thus established arbitrary and flexible virtual filters to reproduce the results that would normally be obtained by taking several acquisitions with real filters.

**Figure 3:**
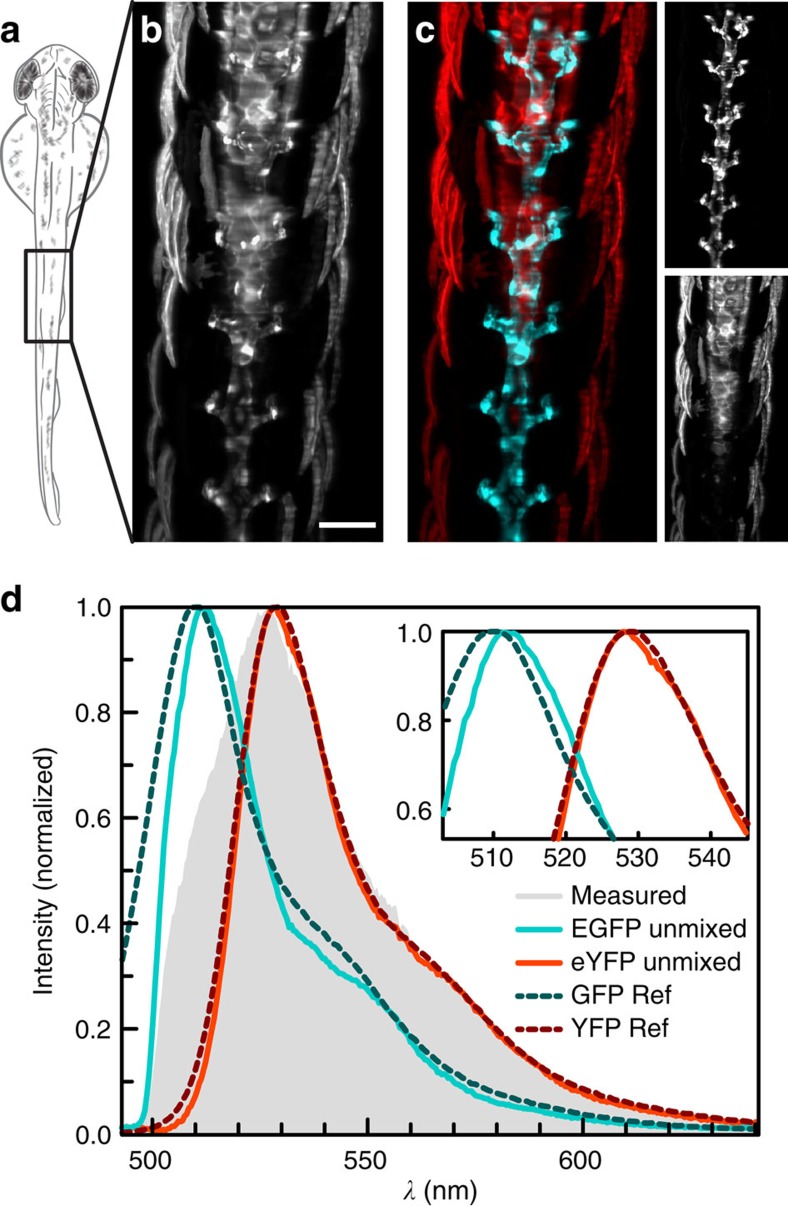
Linear unmixing of EGFP and eYFP. (**a**) Schematic drawing of a two days post fertilization zebrafish embryo imaged dorsally. (**b**) Integration of the reconstructed data set from a *Tg(kdrl:EGFP,s1013t:Gal4,UAS:ChR2-eYFP)* zebrafish along *λ*. Contributions from EGFP and eYFP could not be distinguished. (**c**) With linear unmixing, EGFP (cyan) and eYFP (red) were separated and (**d**) their spectra determined: measured spectrum (grey), EGFP (cyan), eYFP (red). Values from previous studies^16^ (dashed) are plotted for comparison. Maximum intensity projections of seven planes, *z*-spacing 10 μm. Scale bar, 100 μm.

With both our virtual and common filters, however, the spectral region containing the overlap between the two fluorophores is ambiguous and the signal has to be discarded (Fig. 2c, yellow spectral region). For fluorophores with a larger overlap, most of the signal cannot be assigned to either fluorophore and is therefore lost. With advanced data analysis techniques, such as linear unmixing, the spectra of all fluorophores can be determined and their contributions to the signal in each pixel can be calculated, thereby also separating spectrally overlapping dyes^2^. We adapted a blind, non-negative matrix factorization algorithm^17^ to our data format consisting of up to 600 spectral channels. With this algorithm we were able to separate EGFP and enhanced yellow fluorescent protein (eYFP), labelling the vasculature and central nervous system, respectively, in living *Tg(kdrl:EGFP,s1013t:Gal4,UAS:ChR2-eYFP)* zebrafish embryos and restore the lost signal (Fig. 3).

### Determination of autofluorescence

**Figure 4:**
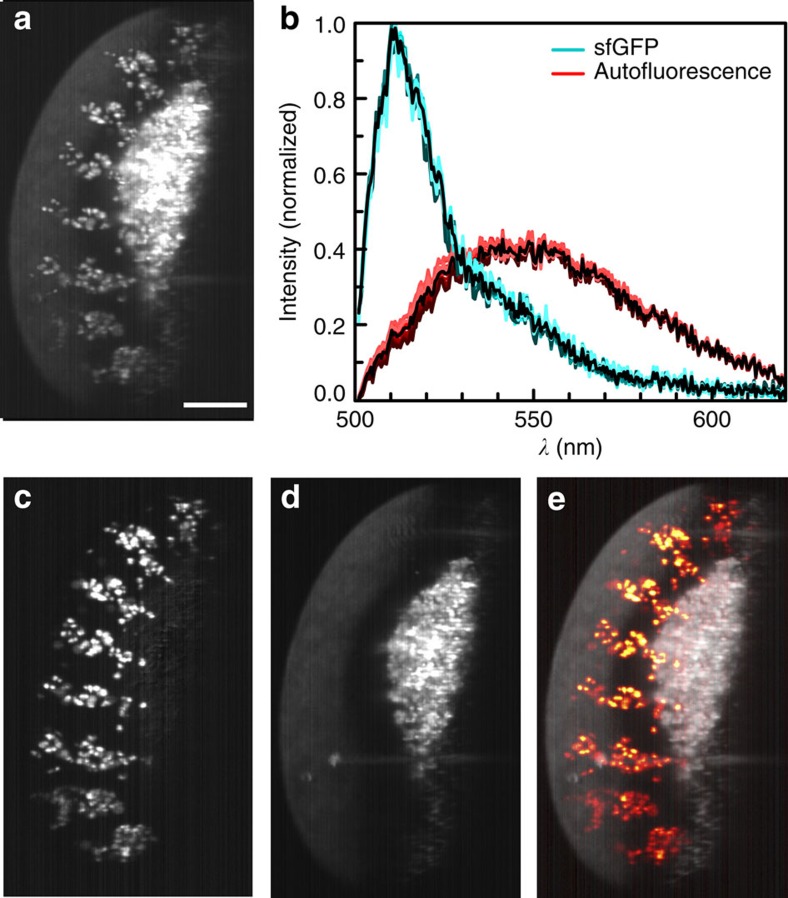
Unmixing of autofluorescence. (**a**) A *λ*-stack of 6 h post-laying *Drosophila* expressing *sens*-sfGFP was integrated between 500 and 570 nm to simulate imaging with a standard GFP bandpass filter. Strong autofluorescence in the green spectral region overlapped with the sfGFP signal. (**b**) Spectra from unmixing: sfGFP (cyan) and autofluorescence (red). Spectra from each plane are plotted in colour, average spectra black. (**c**) After unmixing for two colours, sfGFP was completely separated and (**d**) autofluorescence removed. (**e**) Combination of fluorescence and autofluorescence signals provide additional context, here the outline of the sample. γ-values of the autofluorescence signal were adjusted to 0.1 to make sample outlines better visible. Maximum intensity projections of 13 planes, *z*-spacing 2 μm. Scale bar, 100 μm.

In addition to the signal originating from labelled tissue, most biological samples exhibit intrinsic fluorescence. Since this so-called autofluorescence usually covers a broad spectral range, it overlaps with most fluorophores, adds unwanted signal to all colour channels and can be suppressed only partially with filters. We used our linear unmixing routine to separate autofluorescence and green fluorescence signal from a Drosophila embryo expressing senseless (*sens*) tagged with superfolder GFP (sfGFP) in sensory organ precursors. We precisely determined and removed the autofluorescence from the weak signal of interest (Fig. 4a,b,e). Since autofluorescence is usually emitted by large, continuous structures, we now used autofluorescence as an additional channel to place the fluorescently labelled structures, which are often sparsely distributed, in their morphological context (Fig. 4c–e).

### Optimized acquisition speeds and spectral sampling

While the high spectral sampling of our system produced spectra comparable to the quality of standard spectrometers, the acquisition time of 7 s and data size of 5 GB for a *z*-plane were not acceptable, as they complicated both the routine acquisition of living samples and data analysis. Both acquisition time and data size scale with the number of recorded colour channels and high-resolution spectra might not be needed for most applications.

**Figure 5:**
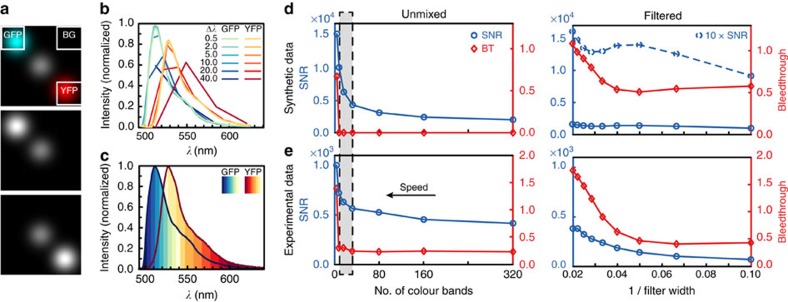
Optimal spectral sampling. (**a**) Synthetic data set consisting of spots exhibiting either EGFP (cyan) or eYFP (red) or the mean of both spectra (white). Spectral sampling, 0.5 nm per pixel. White frames, regions used to determine background (BG), bleedthrough (BT) from the EGFP (GFP) and the eYFP channel (YFP). (**b**) Spectra obtained after downsampling the *λ*-stack to generate larger spectral sampling and unmixing downsampled data; EGFP (blue), eYFP(red). (**c**) Dual channel synthetic data with different filter widths was generated by integration starting at short wavelengths for EGFP (blue) and at long wavelengths for eYFP (red). Hues illustrate intervals used to simulate different filter widths. (**d**) SNR (blue) and BT (red) were determined for the synthetic data set. Hyperspectral, unmixed data shown on the left, dual channel, filtered data on the right. (**e**) The analysis was repeated for the experimental dataset shown in Fig. 3. For increasing filter widths, both SNR and BT increase. For decreasing number of colour bands, both imaging speed and SNR improve but BT remains constant down to 32 bands, thereby defining the optimal number of bands for hyperspectral imaging (highlighted).

We determined the optimal spectral sampling for hyperspectral imaging and compared the performance of hyperspectral imaging and filter-based approaches as described in the Methods section (Fig. 5). For filtered data, image quality (characterized by signal-to-noise ratio (SNR)) increased for broader filter bands. At the same time, however, it became more difficult to discriminate between both colour channels, because the EGFP and eYFP spectra overlap strongly over their entire spectral range. Surprisingly, unmixing performance remained almost constant as the number of spectral channels was decreased and their width was increased, even below a spectral sampling of 5 nm, when spectra were not reproduced faithfully anymore. Only when a width of 40 nm per channel (corresponding to only four spectral channels) was reached, performance decreased steeply and the two colours could not be distinguished anymore (Supplementary Fig. 4 and Supplementary Note 1).

Our results show that a spectral sampling of 20 nm was sufficient to unmix EGFP and eYFP successfully. For environmental sensing applications, however, it might be interesting to utilize the information contained in the spectra, for example, of pH-sensitive dyes. Therefore, we aimed for a spectral sampling that still reproduced spectra reliably. Integration of Specim's Imspector Fast10 provided us with a spectral sampling of 5 nm per pixel, while data size was reduced to one tenth (500 MB), and the acquisition of a single plane required <1.5 s.

### Unmixing of five fluorophores

**Figure 6:**
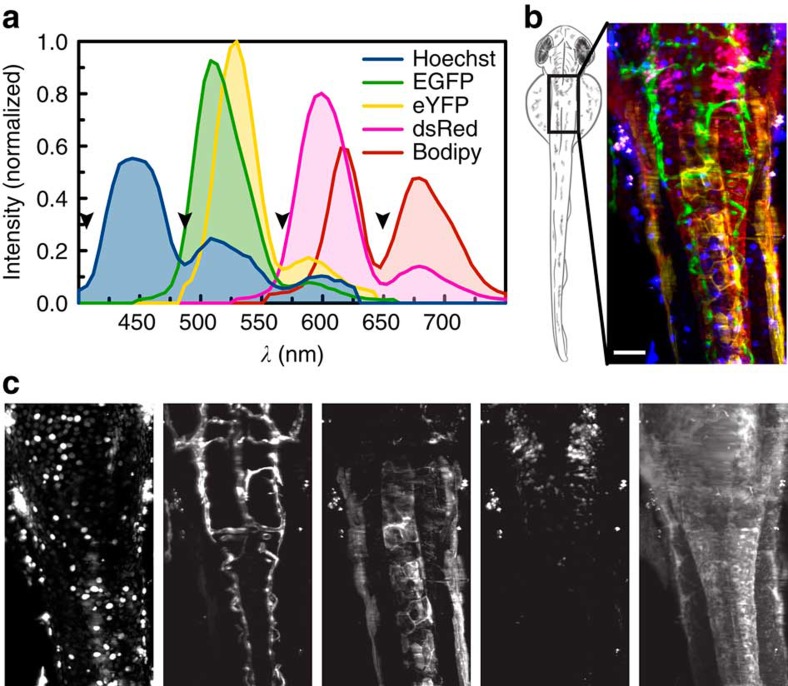
Linear unmixing of five fluorophores. (**a**) For imaging five colours, wild-type fish stained with Hoechst or Bodipy as well as fish expressing only EGFP, eYFP or dsRed were imaged to obtain reference spectra. Black arrowheads highlight regions where the signal is suppressed by the QuadNotch Filter. (**b**) A zebrafish embryo expressing *Tg(kdrl:EGFP,s1013t:Gal4,UAS:ChR2-eYFP,ptf1a:dsRed)* was stained with Hoechst and Bodipy and imaged dorsally. Pseudo-colour overlay after unmixing the hyperspectral data set with reference spectra. For better visualization, Bodipy intensity is reduced to 75%. (**c**) Individual channels, from left to right: Hoechst (blue), EGFP (green), eYFP (yellow), dsRed (magenta) and Bodipy (red). Maximum intensity projections of 65 planes, *z*-spacing 2 μm. Scale bar, 100 μm.

If only two different labels are needed, one can select fluorophores with little spectral overlap that are well separable. When adding more colours to explore the interplay between many tissues in a living organism, the situation becomes more complex and with increasing spectral overlap it becomes impossible to distinguish multiple colours with conventional filters. In particular non-vital dyes exhibit very broad spectra contaminating all channels at higher wavelengths. Having access to the full spectrum, we could now unmix, separate and quantify many markers across the visible range. Here we demonstrate that our unmixing approach worked well for samples labelled with five different fluorophores, for which we stained the nuclei and intracellular membranes of *Tg(kdrl:EGFP,s1013t:Gal4,UAS:ChR2-eYFP,ptf1a:dsRed)* zebrafish embryos with Hoechst and Bodipy, respectively. The overlapping structures could be clearly distinguished after linear unmixing (Fig. 6).

## Discussion

We designed a hyperspectral light sheet microscope based on a scanned illumination and descanned detection. With this optical design, the integration of other scanned illumination schemes, such as two-photon illumination or Bessel beams, will be straightforward. For our setup, we developed two different evaluation workflows to separate the contributions of the recorded fluorophores: one was based on virtual bandpass filtering of the hyperspectral data, which was flexibly adapted depending on the used fluorophores. The other utilized linear unmixing and rendered both the contributing spectra and their intensities for each pixel. We demonstrated that hyperspectral imaging combined with linear unmixing surpassed filter-based approaches in both the image quality and the ability to discriminate between different fluorophores, especially for large spectral overlaps.

We presented two different implementations of the hyperspectral SPIM: the first featured high spectral resolution and produced spectra comparable to data obtained with standard spectrometers. Since such high spectral information content would be superfluous for imaging applications, we implemented a hyperspectral SPIM with reduced spectral sampling. Still, spectra are reproduced faithfully and could potentially be used to monitor environmental conditions by employing fluorophores sensitive to such changes.

With our hyperspectral SPIM, we recorded spectrally resolved data cubes of living samples. By linear unmixing, we separated fluorophores with large spectral overlap and determined their spectra from data sets of multiply labelled zebrafish. For strongly autofluorescent Drosophila embryos, we successfully isolated the autofluorescence and recovered the desired, faint fluorescence signal. The additional information provided by autofluorescence comes for free, as it requires neither extra labelling nor additional excitation.

The hyperspectral SPIM opens up the possibility to simultaneously image dozens of fluorophores expressed in different tissues of a living organism. A single recording may then provide all the information necessary to study the development and interactions of many tissues that is otherwise difficult to assemble from data sets acquired from several individuals. Unfortunately, at current, samples expressing many different fluorophores in distinct structures are still hard to find: commonly, only two or three easy-to-separate fluorophores in the red and green (and maybe blue) colour channel are used. As the collection of samples expressing multiple fluorophores is constantly growing, our hyperspectral imaging technique will serve as a powerful tool to provide a comprehensive atlas of embryonic development.

## Methods

### Setup

A schematic of the hyperspectral SPIM setup can be found in Supplementary Fig. 2 and the optical properties of the components are listed in Supplementary Table 1. The hyperspectral SPIM consisted of two identical water-dipping Nikon CLI75 16x/0.8 objectives that were inserted into the custom-made acrylic sample chamber through openings sealed with O-rings. One objective was used for illumination, one for detection. The sample was inserted from the top and moved with an *xyz* manual translation stage (M-460A-XYZ, Newport, USA) to ensure correct positioning at the intersection of the focal planes of illumination and detection objective. For stack acquisition, the sample was moved in *z*-direction with a motorized linear stage (M111.1DG, Physik Instrumente, Germany).

An LED (CCS TH-27/27-SW, Stemmer imaging GmbH, Germany) was placed opposite of the detection objective for bright field illumination. For light sheet illumination, a multicolour laser light engine (SOLE-6, Omicron-Laserage Laserprodukte GmbH, Germany) with 405 nm (120 mW), 488 nm (200 mW) and 561 nm (150 mW) lasers was used. While scattered laser light could also be removed computationally by discarding the affected wavelengths from the hyperspectral data sets, bright signal might bleed into neighbouring pixels and was therefore suppressed with filters. Depending on the illumination scheme (single wavelength versus multiple wavelengths), scattered laser light was blocked with a 488 LP Edge Basic Filter (Chroma Technology Corporation, USA) or a Quad-Notch Filter 400–410/488/561/631–640 (Semrock, USA). Since several fluorophores could be excited with a single wavelength (for example, GFP and YFP), our illumination scheme with three excitation wavelengths and a Quad-Notch Filter was sufficient for many available fluorophores. A list of laser lines, filters and fluorescent markers used for each presented acquisition can be found in Supplementary Table 2.

In both the detection and illumination arms, a 4*f*-system of tube and scan lens was used to image the objective lenses' back focal plane onto the scan and descan mirrors (dynAXIS M, Scanlab AG, Germany). The mirrors were driven with an analogue output card (NI PCI-6733, National Instruments Corporation, USA). In the illumination arm, the scan mirror was used to form the light sheet, whereas the descanning mirror in the detection arm projected the fluorescence from the illuminated part of the sample onto a single line.

An imaging spectrograph was placed with its entrance slit in the image plane of this line, separating the incoming multicolour signal spatially. Depending on the spectral sampling and acquisition time required, we used either the Imspector V8E (Specim, Finland) for slow acquisitions with a spectral sampling of 0.5 nm per pixel or an Imspector FAST10 for high acquisition speeds with a reduced spectral sampling of 5 nm per pixel. The *x, λ*-image formed by the imaging spectrograph was recorded with an sCMOS camera (Zyla 5.5, Andor, United Kingdom), which was attached to the C-mount of the imaging spectrograph. The overall magnification of the system was determined to be 13.75.

A 45° flip mirror was placed behind the tube lens and a CCD camera (Stingray F-145 B/C, Allied Vision Tech, USA) was used to observe a DSLM-like wide-field image and to facilitate sample positioning. Before measurements, the field of view on the Stingray was checked to match the field of view of the Imspector-Zyla assembly with a bead sample.

### Wavelength calibration

In order to calibrate the recorded wavelengths, the spectrum of each laser line of the SOLE-6 (405, 488, 514, 561, 594 and 638 nm) was recorded once with a spectrometer (USB4000 Miniature Fiber Optic Spectrometer, Ocean Optics, USA). On each day of measurements, all filters were removed and the scattered signal of all six laser lines was recorded simultaneously on the camera chip. The intensities of both measurements were fitted with Gaussian distributions to determine the centre positions using a MATLAB (MathWorks, USA) script. The acquired centre positions were fitted linearly to obtain the calibration curve (Supplementary Fig. 3). This calibration is fast and can be easily repeated on each day of measurements to ensure precise and reproducible spectra.

### Acquisition and online reconstruction of descanned data

During data acquisition, lasers, scan mirrors and motorized stage were controlled via a LabVIEW (National Instruments) interface. To ensure precise synchronization, the camera received a digital trigger when the scan and descan mirrors began moving synchronously to scan the laser through the sample and descan onto the camera, respectively (Supplementary Fig. 5). Laser powers for imaging were set between 2 and 10 mW in the back focal plane of the illumination objective, depending on laser colour and brightness of the fluorescent marker.

For the Imspector VE8, the camera typically acquired a stack consisting of around 2,000 *x*, *λ*-images of 2,000 × 660 pixel (spatial × spectral). With a dynamic range of 16 bit, this amounts to 5 GB of data per acquired *z*-plane. The camera was run at 285 fps, which was the maximum possible frame rate for the chosen image size, amounting to a total acquisition time of 7 s for the whole *λ*-stack. For the Imspector FAST10, only 70 spectral channels were acquired. Therefore, a typical stack consisted of 2,000 *x*, *λ*-images of 2,000 × 70 pixel, amounting to 500 MB per *z*-plane. For this stack size, the camera was run at almost 1,500 fps, reducing the acquisition time to 1.5 s.

A Fiji^18^ plugin using the Java Native Interface for function calls into the Andor Software Development Kit was used to control camera settings and read the images from the camera. The same plugin transformed the acquired *y*-stacks into *λ*-stacks in real time.

### Linear unmixing and data visualization

In order to separate the acquired spectral data according to the contributions of the individual dyes, a Fiji plugin implementing a blind linear unmixing algorithm^17^ was adapted to our data sets. In brief, the algorithm adjusted both the spectra and concentrations for each dye in each pixel iteratively. The update rules were designed to assume a Poissonian distribution of photon counts. When unmixing was completed, a multichannel image and the spectra of each contributing dye were obtained. Fiji was used to visualize the acquired *λ*-stacks and unmixed images. To visualize the obtained spectra, LibreOffice (The Document Foundation) was used. Literature values for the spectra were obtained from the collection on the Tsien-lab homepage^16^.

### Optimal spectral sampling for hyperspectral imaging

In our line-scanning hyperspectral SPIM, acquisition time increased linearly with spectral sampling. Furthermore, high spectral sampling might have adverse effects on image quality, since spreading of the fluorescence signal across many camera lines was expected to reduce SNR. On the other hand, it may no longer be possible to discriminate between different fluorophores with reduced spectral sampling.

We determined the optimal spectral sampling for hyperspectral imaging by calculating image quality and the ability to discriminate different fluorophores (avoiding bleedthrough, BT). We defined the SNR of the whole image area as a measure for image quality in each channel


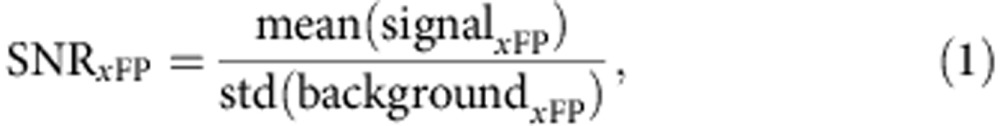


where the pixels containing signal were selected by mean thresholding and the background was determined from an image area not containing any relevant signal; *x*FP denotes fluorescent protein *x* and *y*FP fluorescent protein *y*. As a measure for BT into each channel, we calculated the SNR of regions containing only one fluorophore, SNR_*x*FP,*y*FP_ and determined their ratios


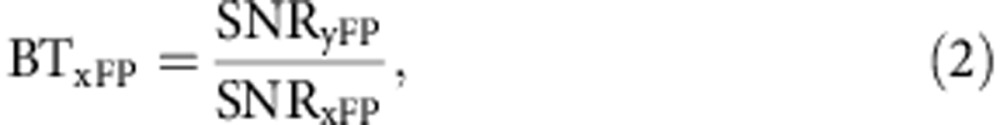


where the subscript *x*FP denotes ‘expected' signal (i. e. GFP signal in the GFP channel) and the subscript *y*FP ‘unexpected' signal (YFP signal in the GFP channel).

Overall SNR and BT were determined as root mean square of the values from each channel


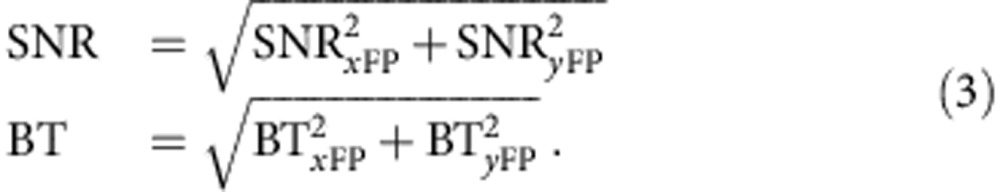


In order to determine the optimal regime, we created a 200 × 200 pixel synthetic data set containing three spots with a Gaussian intensity distribution in MATLAB. One of these spots was multiplied with a GFP spectrum obtained from measurements, one with a YFP spectrum and the third with the mean of both spectra. From this simulated data set with a spectral sampling of 0.5 nm per pixel, we generated data with reduced spectral resolution by integrating across several channels of the *λ*-stack. We unmixed all of these downsampled data sets to obtain separate images for each colour channel. In the same way, data acquired with different filter bands was simulated by integrating the respective bands in the raw data set. To obtain the GFP channel, integration always started from short wavelengths (492 nm), integrating towards longer wavelengths in 5 nm increments, with the first interval covering 10 nm. For YFP, integration was started at long wavelengths (647 nm), integrating towards shorter wavelengths in 10 nm intervals with a first interval of 50 nm. Increments and first intervals where chosen to exclude areas with little change from the analysis (for example in the spectral region above 600 nm, not much change is expected since both spectra run parallel). For each spectral sampling and filter width of the unmixed and filtered data sets, respectively, SNR and BT where calculated. The evaluation workflow is also illustrated in Fig. 5.

### Transgenic lines and sample preparation

Zebrafish (*Danio rerio*) adults and embryos were kept at 28.5 °C and were handled according to established protocols^19^. At 24 h post fertilization, the embryos were treated with 0.2 mM 1-phenyl 2-thiourea (Sigma) to inhibit melanogenesis.

Fluorescent transgenic lines, *Tg(h2afva:h2afva-GFP)*^20^, *Tg(kdrl:Hsa.HRAS-mCherry)*^21^, *Tg(kdrl:EGFP)*^22^ and *Tg(s1013t:Gal4,UAS:ChR2-eYFP)*^23^ were crossed to demonstrate dual-colour acquisition with virtual filters and unmixing of two colours, respectively. For the five-colour imaging, zebrafish expressing *Tg(kdrl:EGFP,s1013t:Gal4,UAS:ChR2-eYFP)* were crossed to zebrafish expressing *Tg(ptf1a:dsRed)*^24^. The embryos were dechorionated manually at the one-cell stage and incubated in 30 μM Hoechst 34580 (Thermo Fisher Scientific) in E3 (ref. 19). Before imaging, Bodipy (Thermo Fisher Scientific) was added to the solution to a final concentration of 1 μM and incubated for 1 h. Before imaging, the samples were washed three times for 5 min with E3.

All zebrafish were imaged at two days post fertilization. During imaging, the samples were anaesthetized with 130 mg l^−1^ Tricaine (Sigma) and embedded in 1.5% low-melting-point agarose (Sigma) inside glass capillaries, according to the protocol described in ref. 25. Agarose embedded zebrafish were extruded from the glass capillary for imaging. The imaging chamber was filled with E3 containing 130 mg l^−1^ Tricaine.

A fosmid^26^ clone containing *sens*^27^ was used to generate a live gene expression marker in *Drosophila melanogaster*. *sens* was tagged C-terminally with superfolder GFP^28^ using liquid culture recombineering^26^. The fosmid clone was inserted into the landingsite VK33 on the third chromosome^29^ using φC31 integrase^30^. A homozygous fly line was established (CD15.1.VK33) with the fosmid transgene being able to recapitulate the endogenous gene expression pattern of *sens*. Embryos were collected at room temperature and bleached for 60 s using 1.4% chlorine for dechorionation and then washed with desalinated water. The embryos were left at room temperature for 6 h post laying until the superfolder GFP signal was visibly expressed. For imaging, the samples were embedded in 1.5% low-melting-point agarose (Sigma) inside glass capillaries and extruded from the capillary. The imaging chamber was filled with PBS.

All animals were treated in accordance with EU directive 2011/63/EU as well as the German Animal Welfare Act.

## Additional information

**How to cite this article:** Jahr, W. *et al.* Hyperspectral light sheet microscopy. *Nat. Commun.* 6:7990 doi: 10.1038/ncomms8990 (2015).

## Supplementary Material

Supplementary Information Supplementary Figures 1-5, Supplementary Tables 1-2, Supplementary Note 1 and Supplementary References.
