# Supplementary material for: Hyperspectral light sheet microscopy
Source: Nat Commun. 2015 Sep 2;6:7990. doi: 10.1038/ncomms8990 (PMC4569691; doi:10.1038/ncomms8990)
Supplement: Supplementary Information — Supplementary Figures 1-5, Supplementary Tables 1-2, Supplementary Note 1 and Supplementary References. [file ncomms8990-s1.pdf]

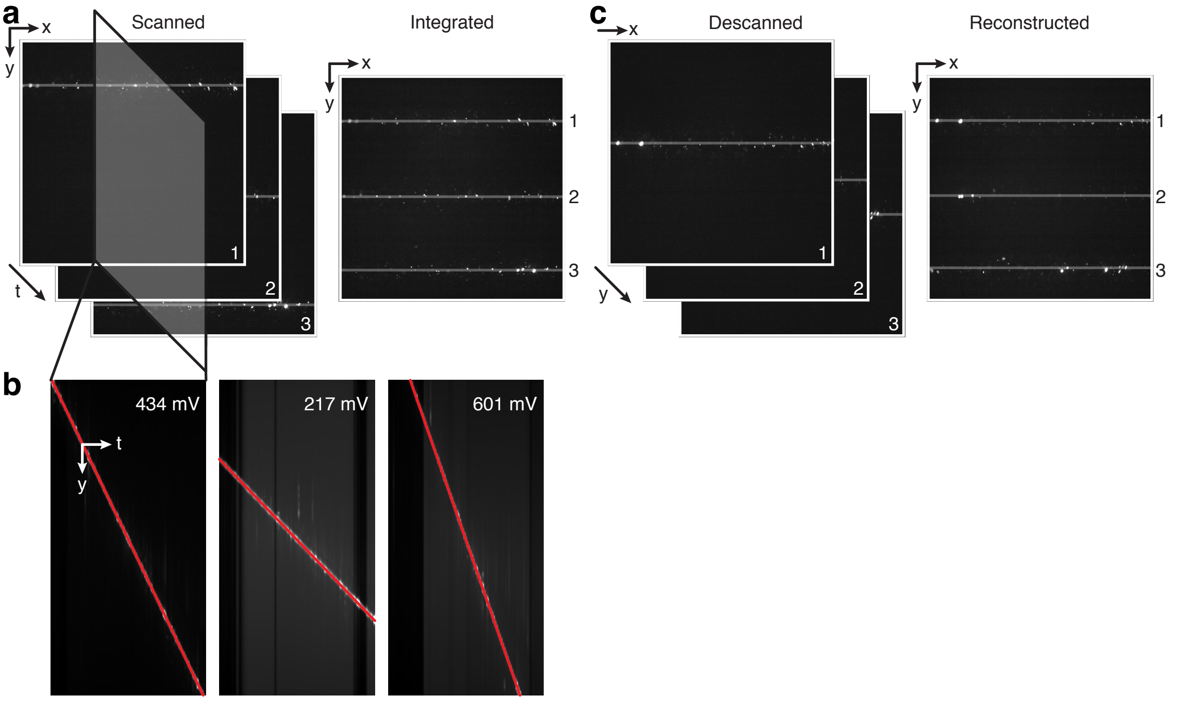

Supplementary Figure 1: **Image formation and calibration of descanned detection.** **(a)** Image formation with a line-scanning SPIM where the laser beam is swept through the sample and different regions are illuminated on the camera chip. An image is formed by integration of the signal, i. e. by adjusting the acquisition time of the camera to capture the whole sweep in one frame. **(b)** For calibration of the scanning process, we did not integrate the signal, but acquired a full frame instead for each laser position. The resulting stack was resliced to display y, t-images and then integrated along x. In the resulting y, t-images, the linearity of the scan process was evaluated to ensure that the used scan lenses did not introduce any non-linearity (red lines). With the resliced images, the scanning voltage (and therefore scanning angle) of the mirrors was adapted until the scanned area covered the whole camera chip. **(c)** In our descanned SPIM, a descanning mirror was introduced into the detection path to project the illuminated region from the sample always onto the centre position of the camera chip. The driving voltage of the descanning mirror was calibrated in the same way as for the scan mirror. In this way, the position of the scanned laser beam in the sample was known for each line acquired and the image was reconstructed line-wise from the descanned data.

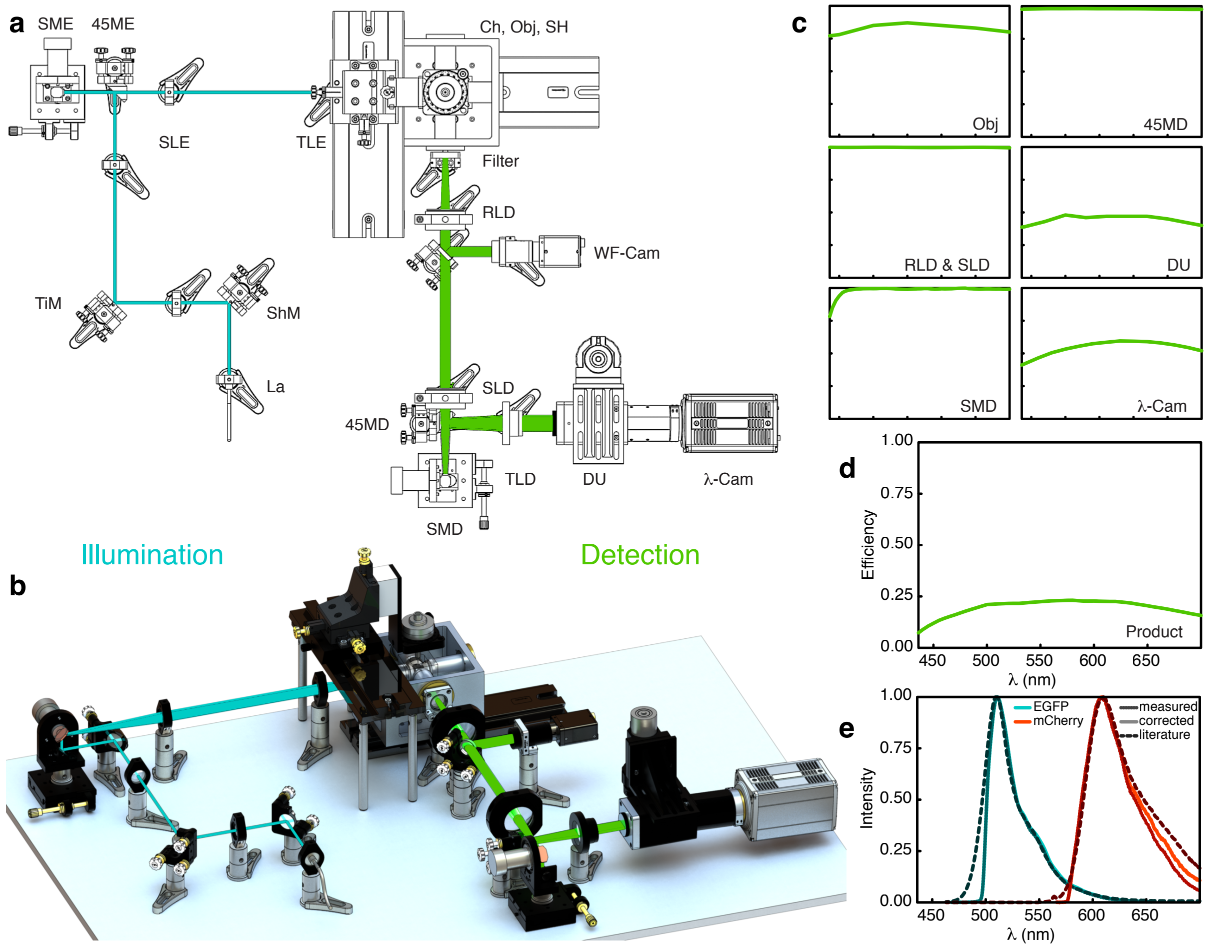

Supplementary Figure 2: **Setup and correction of spectral sensitivity.** **(a)** Technical drawing of the setup. The beam of the laser engine was fibre-coupled into the system (La). Two mirrors in the respective conjugated planes were used to shift (ShM) or tilt (TiM) the light sheet position in the sample. A 45° mirror (45ME) was used to reflect the incident light onto the excitation scan mirror assembly (SME), before imaging the scanned light sheet through a $4f$-system of excitation scan lens (SLE) and tube lens (TLE) into the sample chamber (Ch) with objectives (Obj). The sample holder (SH) with the agarose-embedded sample was inserted from top. The emission filter was placed behind the detection objective. A $4f$-system of a detection relay lens (RLD) and scan lens (SLD) projected the signal onto the detection scan mirror assembly (SMD). Another 45° mirror coupled the light into the detection tube lens (TLD), which imaged the descanned line onto the entrance slit of the defractive unit (DU). The x, λ-image was recorded on the spectral detection camera (λ-Cam). Behind the relay lens, a 45° flip mirror could be used to alternatively image the widefield signal onto a second camera (WF-Cam) for easier sample positioning. The associated list of all components can be found in Supplementary Table 1. **(b)** Computer rendering of the setup. **(c)** In order to determine the spectral efficiency of the hyperspectral SPIM, we obtained the respective curves for each optical element from the manufacturers: transmission curves for lenses, filters and the diffractive unit, reflection curves for the mirrors and quantum efficiency curve for the camera. **(d)** Overall spectral efficiency was calculated by multiplying the individual curves and ranged between 7 and 23 % from 440 to 700 nm. Axes labels in (c) and (d) are identical. **(e)** We used this curve to correct EGFP (cyan) and mCherry (red) spectra measured in transgenic zebrafish and compared the values to spectra obtained from earlier studies [1]. We observed good agreement with the literature for both corrected spectra and uncorrected spectra. Since the spectral efficiency curve of our hyperspectral SPIM was flat, it was not necessary to correct acquired spectra in most cases. Measured values (dotted), corrected values (continuous), values from literature (dashed).

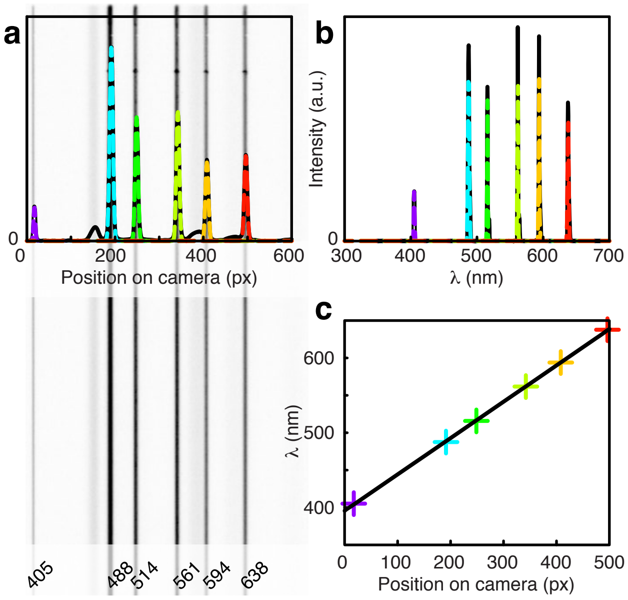

Supplementary Figure 3: **Calibration of wavelength response.** We calibrated the wavelength response of our system on each day before measuring. **(a)** For calibration, all six laser lines (405, 488, 514, 561, 594 and 638 nm) were projected into the water filled chamber (no sample inserted, no filter used). The peak positions on the camera chip were determined by fitting their intensity plot with Gaussians. **(b)** To assign positions on the camera to a wavelength, the laser spectra were determined once with a spectrometer (USB4000 Miniature Fibre Optic Spectrometer, Ocean Optics, USA) and fitted with Gaussians. **(c)** The resulting maxima from both measurements were fitted with a linear function to obtain the calibration curve of the setup.

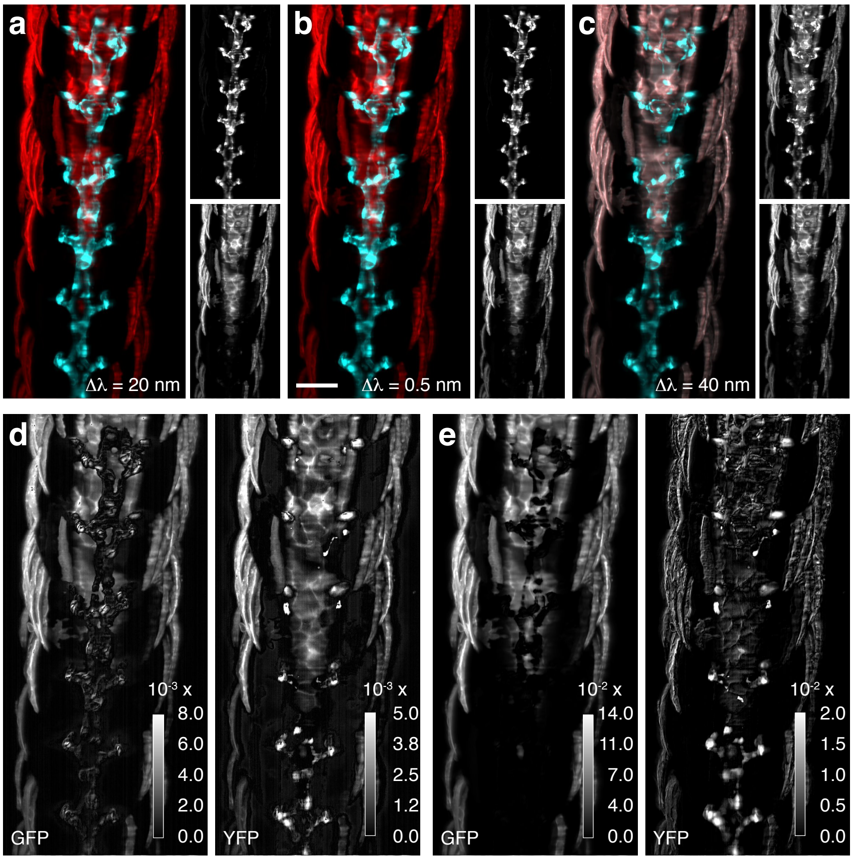

Supplementary Figure 4: **Efficiency of Unmixing.** **(a)** Dual-channel image unmixed from a stack with spectral sampling of 0.5 nm, **(b)** 20 nm and **(c)** 40 nm. EGFP (cyan), eYFP (red). **(d)** StD of unmixing results from a stack with spectral sampling of 0.5 nm and 20 nm. **(e)** StD of unmixing results from a stack with spectral sampling of 0.5 nm and 40 nm. StD between EGFP channels (left) and eYFP (right).

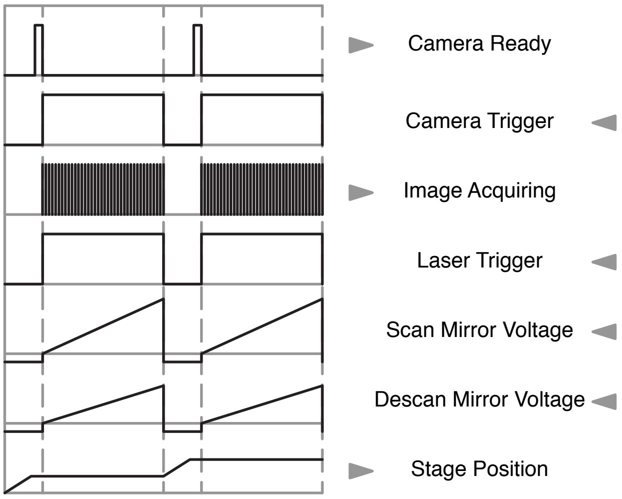

Supplementary Figure 5: **Triggering scheme.** The microscope was controlled with an analogue input output module (NI PCI-6733, National Instruments Corporation, USA) through a custom interface in the LabVIEW environment. A camera-start trigger was sent to the camera, which then acquired all x, λ-images for the y-stack in free run mode. At the same time, the laser sources were triggered and the scan mirror voltages were changed linearly to scan the beam through the sample and descan the acquired line. Once the camera finished acquiring and writing files, a camera-ready signal was sent to the software. For z-stacks, the motorized stage moved stepwise between the acquisitions of each plane. Arrowheads pointing left indicate signals sent from the control PC, arrowheads pointing right signals sent to the PC.

Supplementary Table 1: **Optical Properties of the used components**

Supplementary Fig. 2	Component	f in mm	$\varnothing$ in mm	AR Coating in nm	Manufacturer
Excitation					
La	Laser Fibre				Omicron
ShM	Shift Mirror		25	400 to 700	Thorlabs
BE1	Beam Expander	80	25	400 to 700	Thorlabs
TiM	Tilt Mirror		25	400 to 700	Thorlabs
BE2	Beam Expander	200	25	400 to 700	Thorlabs
45ME	45° Mirror		25	400 to 700	Thorlabs
SME	Scan Mirror		12.7	400 to 700	Thorlabs
			24 × 14.7	Silver	Scanlab
SLE	Scan Lens	150	25	400 to 700	Thorlabs
TLE	Tube Lens	100	25	400 to 700	Thorlabs
Obj	CFI LWD 16x W, NA 0.8, W.D. 3.0 mm	12.5			Nikon
Detection					
Obj	CFI LWD 16x W,	12.5			
	NA 0.8, W.D. 3.0 mm				Nikon
Filter	QuadNotch Filter or		25	405, 488, 561, 638	Semrock
	488 LP Edge		25	488	Chroma
RLD	Relay Lens	150	50	400 to 700	Thorlabs
SLD	Scan Lens	100	50	400 to 700	Thorlabs
SMD	Scan Mirror		24 × 14.7	Silver	Scanlab
			25, elliptic	400 to 700	Thorlabs
45MD	45° Mirror		25	400 to 700	Thorlabs
TLD	Tube Lens	200			Nikon
DU	Diffractive Unit				Specim
λ-Cam	Zyla 5.5				Andor
WF-Cam	Stingray F145 B/C				AVT

Supplementary Table 2: **Excitation wavelengths, fluorophores and filters used for each dataset.** Depending on the fluorophores present in the sample, different combinations of excitation lasers and filters were used. In short, if one laser line was sufficient to excite all fluorophores present (i. e. for samples expressing only one fluorophore or for combinations of EGFP and eYFP), a 488 LP Edge filter was used. If several laser lines were needed to excite all fluorophores, a Quad-Notch filter was used to suppress scattered excitation light.

figure	wavelength	fluorophores	filter
Fig. 1	488 nm	YFP	488 LP Edge
Fig. 2	488 and 561 nm	GFP, mCherry	Quad-Notch
Fig. 3	488 nm	GFP, YFP	488 LP Edge
Fig. 4	488 nm	GFP	488 LP Edge
Fig. 6	405, 488 and 561 nm	Hoechst, GFP, YFP, Bodipy	Quad-Notch

Supplementary Note 1: Efficiency of Unmixing

We determined the minimal spectral sampling needed with our setup and data processing routine to unmix EGFP and eYFP reliably. The overall results are shown in the main text (Fig. 5). Here, we discuss more specific results on the efficiency of linear unmixing with the help of the dual-colour dataset shown in Fig. 3.

The dataset was originally acquired with a spectral sampling of $0.5\,\mathrm{nm\,px}^{-1}$ and then downsampled by integrating over colour bands of different widths. We unmixed all downsampled datasets to obtain dual-colour images. The unmixed images from λ-stacks with 0.5, 20 and 40 nm spectral sampling are shown in Supplementary Fig. 4a-c. When comparing the dual-colour images for $\Delta\lambda = 0.5\,\mathrm{nm}$ and $\Delta\lambda = 20\,\mathrm{nm}$, we did not observe any differences. For $\Delta\lambda = 40\,\mathrm{nm}$, however, EGFP and eYFP could not be unmixed anymore. Especially in the EGFP channel, we observed bright contributions from the eYFP labelled structures.

In order to quantify these differences, we normalized each channel in all dual-colour images to values between 0 and 1 using Fiji [2]. For each pixel in these images, we calculated the standard deviation (StD) and assigned grey scale values to the StD for visualization. Supplementary Fig. 4d illustrates the StD of the EGFP channel (left) and eYFP channel (right) unmixed from λ-stacks with 0.5 and 20 nm spectral sampling. In the StD map for the EGFP channel, we observed bright signal where contributions from eYFP labelled structures bled into the EGFP channel. EGFP labelled tissue appeared as a negative imprint of low StD, implying that EGFP was unmixed equally well for both low and high spectral sampling.

We also observed the bleedthrough of eYFP into the green channel in the StD map for the eYFP channel, where eYFP labelled structures left a bright imprint. Additionally, high StD spots corresponding to EGFP bleeding into the eYFP channel were observed. Overall, these deviations were less than 1 % in both channels and intensities were therefore too low to be observed when comparing the dual-colour images directly.

The StD maps obtained from the comparison between dual-colour images unmixed from 0.5 and 40 nm spectral sampling yielded similar results. In the EGFP and eYFP StD maps, strong contributions from the other channel were evident (Supplementary Fig. 4e). However, bleedthrough was much larger: For EGFP, it was up to 2 % and up to 14 % for eYFP. Therefore, eYFP contributions could easily be observed when comparing the dual-colour images.

The results discussed here agree well with the SNR and BT curves shown in the main text: As the spectral sampling was decreased, BT remained constant down to $\Delta\lambda = 20\,\mathrm{nm}$ and increased steeply for smaller spectral sampling. Furthermore, the StD of less than 1 % observed for $\Delta\lambda = 20\,\mathrm{nm}$ could easily be ignored when optimizing the imaging system for speed.

Supplementary References

[1] Tsien, R. Y. Fluorophore reference spectra. Accessed Jan 13, 2015, http://www.tsienlab.ucsd.edu/Documents.htm.

[2] Schindelin, J. *et al.* Fiji: an open-source platform for biological-image analysis. *Nat. Methods* **9**, 676–682 (2012).
